# Platelet‐rich plasma and regenerative dentistry

**DOI:** 10.1111/adj.12754

**Published:** 2020-03-24

**Authors:** J Xu, L Gou, P Zhang, H Li, S Qiu

**Affiliations:** ^1^ Shenzhen Longgang Institute of Stomatology Shenzhen Guangdong China; ^2^ Department of Otolaryngology Longgang E.N.T. Hospital & Shenzhen Key Laboratory of E.N.T. Institute of E.N.T Shenzhen Guangdong China; ^3^ Center for Genetic Medicine Xuzhou Maternity and Child Health Care Hospital Xuzhou Jiangsu China

**Keywords:** Cytokines, growth factors, platelet‐rich plasma, regenerative dentistry

## Abstract

Regenerative dentistry is an emerging field of medicine involving stem cell technology, tissue engineering and dental science. It exploits biological mechanisms to regenerate damaged oral tissues and restore their functions. Platelet‐rich plasma (PRP) is a biological product that is defined as the portion of plasma fraction of autologous blood with a platelet concentration above that of the original whole blood. A super‐mixture of key cytokines and growth factors is present in platelet granules. Thus, the application of PRP has gained unprecedented attention in regenerative medicine. The rationale underlies the utilization of PRP is that it acts as a biomaterial to deliver critical growth factors and cytokines from platelet granules to the targeted area, thus promoting regeneration in a variety of tissues. Based on enhanced understanding of cell signalling and growth factor biology, researchers have begun to use PRP treatment as a novel method to regenerate damaged tissues, including liver, bone, cartilage, tendon and dental pulp. To enable better understanding of the regenerative effects of PRP in dentistry, this review describes different methods of preparation and application of this biological product, and provides detailed explanations of the controversies and future prospects related to the use of PRP in dental regenerative medicine.

**Abbreviations and acronyms**
EGF: epidermal growth factor
FGF: fibroblast growth factor
HGF: hepatocyte growth factor
IGF‐1 and IGF‐2: insulin‐like growth factors 1 and 2
PDGF: platelet‐derived growth factor
PRP: platelet‐rich plasma
VEGF: vascular endothelial growth factor

## INTRODUCTION

### Platelet‐rich plasma

Platelet‐rich plasma (PRP) is extensively applied as a bioactive scaffold in cell‐based therapy and tissue engineering. It is prepared from autologous plasma with concentrated platelets.^1^ Notably, platelets contain more than 300 biologically active molecules that are released upon activation from platelet alpha and dense granules and subsequently regulate the tissue regeneration process.^2^, ^3^ Activated platelet‐derived factors serve as messengers and regulators that influence a variety of cell–cell and cell–extracellular matrix (ECM) interactions and serve to modify the pericellular microenvironment.^4^, ^5^, ^6^ The most important growth factors released by platelets in PRP include vascular endothelial growth factor (VEGF), transforming growth factor‐β (TGF‐β), platelet‐derived growth factor (PDGF), fibroblast growth factor (FGF), epidermal growth factor (EGF), hepatocyte growth factor (HGF), insulin‐like growth factors 1 and 2 (IGF‐1 and IGF‐2), matrix metalloproteinases 2 and 9, and interleukin‐8.^7^, ^8^ These bioactive molecules play important roles in different applications of regenerative medicine, including bone remodelling, wound healing, hair regrowth, nerve regeneration, aging facial skin, acne scarring, androgenic alopecia and diabetic wounds. During recent years, growing studies have investigated the application of PRP in regeneration of different tissues. This review addresses the different methods of preparation of PRP and clinical applications of this biological product in regenerative dentistry.

### Application of PRP in dental regenerative medicine

In recent years, PRP has been extensively investigated in regenerative medicine. It contains growth factors that influence wound healing, such that it can greatly contribute to tissue repair. In surgery, PRP reduces bleeding while enhancing soft tissue healing and bone regeneration. Moreover, the cost of regeneration therapy can be reduced using PRP.^9^ Thus, PRP has gained increasing popularity in the medical field, especially in regenerative dentistry, including regenerative endodontics (pulpotomy, apical surgery and apexification), periodontics (treatment of infrabony periodontal defects and periodontal plastic surgery), and oral and maxillofacial surgery (tooth extractions, soft tissue and bone tissue surgery, and implant surgery).^10^, ^11^, ^12^ PRP has been used in many dental regeneration procedures and has yielded promising results.

### PRP in endodontic healing

Regenerative endodontics aims to regenerate damaged/necrotic pulp–dentin complex tissues such as dentin, pulp tissue and root structures, in order to restore pulp functions. After proper disinfection, pulp‐like tissue may be potentially formed by a variety of pre‐existing dental stem cells in the presence of suitable growth factors and scaffold medium. Autologous PRP has been widely used in several branches of dentistry because of its ability to release a rich source of healing‐promoting growth factors that favour stem cell multiplication and differentiation, and because of its ability to act as an ideal three‐dimensional scaffold medium.^12^, ^13^ PRP has recently emerged as a possible tool for supporting cell growth and differentiation of vital tissues in the canal after disinfection, thereby enhancing endodontic regeneration.^14^ Indeed, the minimally invasive technique of revascularization is an effective treatment modality for management of immature permanent teeth with compromised structural integrity.^15^

There have been multiple case reports of successful single‐visit regenerative endodontic therapy involving the use of PRP. A single‐visit revascularization procedure has two advantages: it reduces the possibility of further bacterial contamination of the root canal and reduces the negative consequences of poor patient compliance with regular follow‐up evaluation.^16^ Although there has been a dramatic increase in the number of published case reports involving endodontic therapy with PRP, a standardized protocol has not yet been established.^16^ Sachdeva *et al*. reported the case of a 16‐year‐old male patient who had an incompletely developed root with an open apex in his discoloured, non‐vital maxillary left lateral incisor. Autologous PRP was injected into the canal space up to the level of the cementoenamel junction, and white mineral trioxide aggregate was placed directly over the PRP clot. The tooth was restored with permanent filling materials 2 days later. Three‐year follow‐up radiography revealed the recovery of the periapical lesion, increased thickening of the root walls, further root development and continued apical closure of the root apex.^17^ Alagl *et al*. explored the effect of PRP in healing and regeneration of pulpal and periapical tissues. They found that PRP can serve as a successful scaffold for regenerative endodontic treatment. With the exception of a significant increase in root length, the results of treatment with PRP were not significantly different from those of the conventional protocol in which a blood clot was used as the scaffold.^18^ Other researchers have also used PRP as a scaffold in regenerative endodontic treatment of necrotic immature teeth, as an alternative to the blood clot scaffold technique.^19^ Regenerative endodontic procedures comprise two separate clinical concepts. The first concept involves a revitalization approach to achieve tissue regeneration, as described above. The second concept involves the active pursuit of pulp and dentin regeneration through the use of tissue engineering technology. Although this technology is in early stages of development, it is expected to allow immature pulpless teeth to continue growth and maturation. Additional translational research is needed to optimize this technology.^20^

There is a considerable lack of standardization in PRP treatment protocols and long‐term high‐quality clinical trials of PRP in the endodontic field. Adequate methodological tools to assess case reports and case series also remain elusive.^12^ Overall, further translational studies are needed to investigate the outcome of applying PRP in regenerative endodontic treatment of immature necrotic teeth and achieve more pulp‐like and dentin‐like tissues in the root canal system; the results of such studies will be essential for establishment of a standardized treatment protocol.

### PRP in periodontal regeneration procedures

Regeneration of tooth‐supporting structures destroyed by periodontitis is a major goal of periodontal therapy.^21^, ^22^ Periodontal regenerative surgery aims to regenerate alveolar bone, cementum and functional periodontal ligament.^23^ PRP is assumed to increase the predictability of periodontal regeneration procedures. When platelets are activated, they exocytose their internal granules; this process is mediated by molecular mechanisms homologous to those of other secretory cells, uniquely coupled to cellular activation through intracellular signalling events.^23^ Growth factors are subsequently released from platelet granules and contribute to chemotaxis, differentiation, mitogenesis and metabolism of cells involved in wound healing.^24^ During periodontal wound healing after the exogenous application of PRP, the delivery of autologous platelets to periodontal wounds increases the local concentrations of growth factors, which then exert regulatory effects on the homeostasis of periodontal tissues and modify the responses of periodontal soft and hard tissues to enhance healing outcomes.^25^, ^26^

One important criterion for periodontal regeneration is the maintenance of a wound space to which periodontal ligament cells can migrate. For the growth factors released by platelets in PRP to exert their potential, a medium that provides this space is needed. Guided tissue regeneration using barrier membranes – a therapeutic modality currently available for periodontal regeneration – provides sufficient space for the migration of periodontal ligament cells and prevents the formation of long junctional epithelium.^27^ Because PRP possesses limited space‐provision potential, it has been used mainly in combination with bone grafts or substitutes.^28^ As demonstrated in a variety of published systematic reviews, the therapeutic efficacy of PRP in periodontal regenerative procedures has produced controversial outcomes ranging from significant to null effects, despite previous evidence of its clinical benefits.^29^, ^30^ Bhardwaj *et al*.^31^ reported that the addition of PRP to bone graft appeared to be beneficial in the treatment of human periodontal intrabony defects. In another case report, the authors treated intrabony defects by adding PRP to a bone allograft in guided tissue regeneration and observed significant improvements in clinical insertion and bone filling.^32^ Regarding the effectiveness of PRP for periodontal regeneration in the treatment of intrabony defects, a meta‐analysis concluded that PRP might offer some beneficial effects with respect to clinical and radiographic outcomes.^33^ A subsequent systematic review evaluated the effect of PRP in various regenerative procedures related to periodontal defects and gingival recession; it concluded that PRP may be advantageously used as an adjunct to graft treatments for intrabony defects, but not for gingival recession.^34^ However, the addition of PRP to bone graft materials has not increased significantly the positive outcomes independent of the type of barrier or graft. The polypeptide proteins of the platelet‐rich plasma do not enhance the clinical regenerative effect of enamel matrix proteins.^35^ Camargo *et al*. compared the results of bovine porous bone mineral and guided tissue regeneration with and without the addition of PRP. They evaluated changes in probing depth (PD), clinical attachment level (CAL) and defect filling at 6‐month follow‐up, and concluded that PRP treatment provided no statistically significant resolution of intrabony defects.^36^ Possible explanations for the discrepancies among outcomes might be related to differences in treatment courses, graft materials and initial clinical parameters.^37^

PRP might become a routine treatment modality for periodontal regeneration in the future because of its advantages.^38^ Long‐term investigations with a large sample size should be performed to further explore the effect of PRP in the management of periodontal regeneration procedures and to verify the results of *in vitro* studies in clinical evaluations.

### PRP in oral and maxillofacial surgery

The regenerative potential of PRP has been explored in considerable depth during the last two decades. The primary goal of using PRP in oral surgery is to regenerate new tissues during the healing process. It has been suggested that platelets in PRP release a pool of growth factors that recruit reparative cells and promote several biological processes necessary for soft tissue repair and alveolar bone regeneration.^39^ Moreover, PRP is autologous and relatively easy to prepare in a dental clinic.^40^ The use of PRP is therefore opening new avenues in the field of tissue repair and regeneration in oral surgery.

A number of studies have shown that PRP gel can significantly reduce postoperative pain and discomfort after tooth avulsion, and can avoid the development of osteitis.^41^ Alissa *et al*.^42^ evaluated the effect of PRP on the healing of extraction sockets. The findings of their studies suggested that postoperative pain was significantly reduced with clinically appreciable soft tissue healing in patients treated with PRP, compared with the control group. In a study of patients who had undergone third molar extraction, Ogundipe *et al*.^43^ showed that treatment with PRP led to pain reduction, as well as improvement in swelling and mouth opening. Ruktowski *et al*.^44^ showed that there was a significant increase in radiographic density over the baseline level after tooth extraction. Prataap *et al*. reported that autologous PRP is a biocompatible material that improves soft tissue healing, reduces pain and decreases the incidence of alveolar osteitis in the extraction socket.^45^ In these studies, the exact definition of PRP products tested is unclear, due to the lack of consistent terminology and associated misunderstandings. However, careful evaluation of the published data reveals that leukocyte‐and‐platelet‐rich plasma (L‐PRP) and pure platelet‐rich plasma (P‐PRP) gels are probably the most frequent products tested in oral and maxillofacial surgery.^41^

PRP‐associated products have been studied *in vitro* and *in vivo* in both maxillofacial surgery and general surgery, and are currently used for various orthopaedic applications.^46^ Clinical researchers recommend the use of PRP prior to or in conjunction with dental implant placement to increase the rate and quality of bone deposition regeneration.^47^ When used as part of a simple and safe therapeutic approach, PRP shows promise in numerous studies in oral and maxillofacial surgery.

### PRP in implant dentistry

PRP has been utilized in dental implantology for stimulating new bone formation or peripheral nerve regeneration. Several animal and human studies have assessed the effect of PRP in implantology. Many of these studies have reported the beneficial effects of PRP on hard and soft tissue healing. For instance, increased bone activity and faster bone regeneration after using PRP were explored by scintigraphy in dogs.^48^ Song *et al*. transferred autologous PRP into the canine implant bed to study the effect of PRP on nerve innervation in the peri‐implant bone. They demonstrated that PRP exhibited a significant effect on the diameter of the myelinated nerve fibres and might help to improve regeneration of nerve fibres in peri‐implant bone, more specifically 6 months after healing.^49^ Taschieri *et al*. reported that the use of PRP in association with implants immediately placed into fresh extraction sockets proved beneficial effects on soft tissue healing in the clinical studies of post‐extraction implants.^50^ Based on the current findings, local application of PRP may provide accelerated healing of hard and soft tissues in the proximity of dental implants during routine implant surgery. However, characterization of the healing with autologous PRP in physiological osseointegration of implants remains poorly documented or even controversial. Moreover, the effect of the concentrations of PRP on the assumed development of peri‐implant bone microstructures in a longer observation time has hardly been investigated.^51^ Further studies should be performed to develop a standardized application of PRP in the field of implant dentistry.

### PRP in sinus‐floor augmentation and bone remodelling

PRP has been added to graft materials including autologous bone, freeze‐dried bone allograft or deproteinized bovine bone mineral for sinus‐floor augmentation.^52^ The beneficial use of PRP as an adjunct to bone substitute material for sinus‐floor augmentation is controversial.^53^ In fact, some articles reported significant advantages of adding PRP to autologous bone or freeze‐dried bone allograft such as certain bone regeneration potential or enhanced bone‐formation rate during sinus‐floor augmentation. Torres *et al*. reported that PRP can improve the regenerative potential of an organic bovine bone by increasing newly formed bone volume.^54^ Similar results were reported by Stumbras *et al*. that PRP combined together with bone graft materials effectively enhanced bone formation and vascularization in maxillary sinus floor elevation. The study suggested that PRP might accelerate bone regeneration by promoting angiogenesis.^55^ However, several clinical reports conducted no beneficial effects of autologous PRP on bone regeneration and formation during maxillary sinus floor elevation. Nikolidakis *et al*. demonstrated that adding PRP to beta‐tricalcium phosphate graft substitute did not provide additional contributions to new bone formation.^56^ Kilic *et al*. evaluated and compared the long‐term clinical and radiographic outcomes between beta‐tricalcium phosphate and PRP, and found that it did not produce significantly more vertical bone height gain or significantly less vertical bone graft resorption compared with graft substitute alone.^57^

### PRP preparation

The clinical application of PRP is based on the high concentrations of growth factors that are released from alpha granules of the concentrated platelets and the secretion of proteins that can exploit the healing process at the cellular level. The quality and functionality of platelets are highly dependent on the protocol used to prepare PRP. Numerous attempts have been made to standardize PRP preparation protocols; however, there is wide variation among studies with respect to the rotational velocity, centrifugation time, blood volume and anticoagulant platelet agonist, such that it is difficult to directly compare the results of these proposed protocols.^58^

PRP can be prepared via one‐step or two‐step centrifugation procedures.^59^ The most frequently cited one‐step protocol is the Anitua plasma‐rich growth factor protocol, which produces a PRP suspension with minimal leukocytes and a reduced platelet concentration, relative to that produced by other PRP preparations.^60^ In the most common two‐step protocol, whole blood is centrifuged at a constant rate to form three layers: a bottom layer containing red blood cells, a central ‘buffy layer’ containing white blood cells, and a top layer containing platelets suspended in plasma. The top layer and part or all of the buffy layer (depending on the leukocyte fraction preferred in the final isolate) are then transferred to a new tube and centrifuged again, resulting in a pellet of platelets.^61^ The constant centrifugation rate is a key step in the preparation of PRP, allowing an increase in the platelet concentration.^62^ Attention must be paid to the use of appropriate centrifugation force to avoid damage to the fragile platelets.^63^ Several methods are currently available for preparation of PRP shown in Table 1. Arora *et al*. studied the platelet concentration through different double centrifugation protocols. The group noticed that, when the centrifugation time increased, the platelet concentration became higher. The highest platelet concentration was obtained by first centrifugation at 440 *g* for 20 min. Moreover, when the group investigated the release rate of different growth factors, they concluded that the best protocol was 208 *g* for 20 min because the increasing centrifugation force led to a platelet aggregation and a bad release of growth factors.^64^ Eren’s article revealed the centrifugation time effect on the growth factors release, using 2660 rpm for 10 min or 12 min. Long centrifugation time significantly increased VEGF concentration, but had no effect on other growth factors.^65^ Amable *et al*. checked up a double centrifugation for platelet isolation and quantification of cytokines and growth factors on 22 healthy subjects. The best performance for platelet concentration is 300 *g* for 5 min in 1st spin. The second centrifugation of 700 *g* for 17 min was chosen for a lower platelet loss.^66^ The optimal condition for obtaining the highest platelet concentration determined by Kahn *et al*. was centrifugal acceleration of 3731 *g* for 4 min using a sample of 478 mL of whole blood.^67^ Slichter and Harker investigated that the highest platelet recovery efficiency was 80% using a sample of 250–450 mL of whole blood centrifuged at 1000 *g* for a period of 9 min.^68^ Bausset *et al*. set up a double concentration protocol and found that 130 or 250 *g* for a period of 15 min was optimal to obtain a platelet concentration factor of 3.47 from the 8.5 mL whole blood.^69^ Yin *et al*. collected peripheral blood from 80 volunteer donors to investigate an optimal double centrifugation. Blood samples were run by choosing relative centrifugal forces (RCF) from 110 to 450 *g*, time from 10 to 15 min. Results showed that 160 *g* for 10 min and 250 *g* for 15 min were designated as the optimal centrifugation conditions for the first and second spins, respectively.^70^ In his study, Franco *et al*. obtained a platelet concentration of 8.5 times higher (603 × 10^3^/µL) by carrying out a first centrifugation at 400 *g* for 10 min and 800 *g* for 10 min for the second spin of the buffy coat.^71^ Mazzocca *et al*. analysed three protocols for preparing PRP samples by studying with eight healthy subjects, a low‐speed centrifugation (1500 rpm for 5 min), a high‐speed centrifugation (3200 rpm for 15 min) and a double centrifugation with a soft spin of 1500 rpm for 5 min and a hard spin of 6300 rpm for 20 min. A high platelet concentration with one centrifugation step at 3200 rpm for 15 min was noted compared with the low speed or double spins.^72^ Landesberg *et al*. utilized 5 mL of whole blood for two spins at 200 *g* for 10 min per spin and obtained PRP samples that had approximately 3.2 times the concentration of the whole blood baseline.^73^ A simple and reproducible PRP preparation method was developed by Rutkowski *et al*. in 2008. The centrifugation step of high‐quality PRP without platelet alteration was 1350 *g* for 10 min.^74^ Jo *et al*. examined the best protocol of the centrifugation time and gravitational force on the platelet recovery ratio of PRP with 39 samples of healthy subjects. Two‐step centrifugation for preparing PRP was used. The first centrifugation was evaluated from 500 to 1900 *g* for 5 min and, from 100 to 1300 *g* for 10 min. In the step 2, platelets in the separated plasma were concentrated at 1000 *g* for 15 min, 1500 *g* for 15 min, 2000 *g* for 5 min and 3000 *g* for 5 min. The maximum efficiency for the first centrifugation step was obtained by applying 900 *g* for 5 min. They achieved 92% efficiency by performing 1500 *g* for 15 min for the second centrifugation step.^75^

**Table 1:** Various protocols for preparation of PRP

Author of study	1st centrifugation	2nd centrifugation	Concentration of platelet
Arora *et al*.^64^	208 *g* for 20 min	1552 *g* for 23 min	87% recovery
Eren *et al*.^65^	2660 rpm for 12 min	–	214.7 ± 52.1 × 10^3^/µL
Amable *et al*.^66^	300 *g* for 5 min	700 *g* for 17 min	140 to 190 × 10^3^/µL
Kahn *et al*.^67^	3731 *g* for 4 min	–	95% recovery
Slichter and Harker^68^	1000 *g* for 9 min	3000 *g* for 20 min	80% recovery
Bausset *et al*.^69^	130 *g* for 15 min	250 *g* for 15 min	3.96‐fold
Yin *et al*.^70^	160 *g* for 10 min	250 *g* for 15 min	1250 × 10^3^/µL
Franco *et al*.^71^	400 *g* for 10 min	800 *g* for 10 min	603 × 10^3^/µL
Mazzocca *et al*.^72^	3200 rpm for 15 min	–	873.8 ± 207.2 × 10^3^/µL
Landesberg *et al*.^73^	200 *g* for 10 min	200 *g* for 10 min	3.2‐fold
Rutkowski *et al*.^74^	1350 *g* for 10 min	–	6‐fold
Jo *et al*.^75^	900 *g* for 5 min	1500 *g* for 15 min	633.2 ± 91.6 × 10^3^/µL

Notably, platelets can be activated before the application of PRP to the target tissue. Thus far, there has been no consensus with respect to whether platelets should be activated prior to their application; there also remains no consensus with respect to the identity of the activating agonist.^76^ Thrombin and calcium chloride (CaCl_2_), which are aggregation inducers, are used with the aim of activating platelets and stimulating degranulation, eventually triggering the release of growth factors.^77^ In the Amable survey, platelet activation was induced by adding 20 mM CaCl_2_ and 25 IU/ml human thrombin incubated at 37 °C for 1 h or at 4 °C for 16 h. The group reported that the resulting platelet product was proven to be rich in platelet‐derived growth factor, endothelial growth factor and transforming growth factor, together with anti‐inflammatory and pro‐inflammatory interleukins.^66^ The thawing frozen platelets as mechanical method was reported to activate growth factors from platelets.^78^ Steller *et al*. compared non‐Ca^2+^‐activated PRP, Ca^2+^‐activated PRP, thawing frozen incubation method on the release of growth factors and investigated the VEGF, PDGF‐BB and TGF‐β1 contents. They concluded that the thawing frozen method is sufficient for releasing growth factors, and calcium activation is not necessary ^79^.

Besides the centrifugation step and activation methods, the volume of processed whole blood and distance from the axis of the centrifugal rotor are other important factors involved in PRP variability.^80^ Perez *et al*. used 3.5 mL of blood to obtain 70–80% platelet recovery and 5 × platelet concentration. However, the recovery of platelets was reduced for a larger blood volume (8.5 mL) processed. As the distances between the surface of whole blood and the rotor were 4.9 cm and 3.0 cm for the processed volumes 3.5 and 8.5 mL, respectively, the mean centrifugal force applied on the erythrocytes decreased with the smaller mean distance from the rotor for the larger volume (8.5 mL) processed under the same angular velocity.^81^ For the commercial PRP preparation systems which are applied in clinical fields, the devices including Curasan, PCCS, Anitus, SmartPReP, GPS, PDGF‐Endoret Kit and the Symphony II system are designed to produce around 6 mL of PRP from 36 to 60 mL of whole blood.^82^, ^83^, ^84^

The concentration of platelets in recovered PRP reportedly ranges up to 1900 × 10^3^/μl.^85^ Some protocols are designed to concentrate platelets 3–9 times, thereby producing a final product with even greater concentrations of growth factors.^86^ The concentrations of PRP components are of utmost importance, as the mechanism of action of PRP is primarily based on the growth factors and cytokines present in the platelet alpha granules.^87^ However, there have been some controversies with respect to this issue because high platelet concentrations in PRP are achieved by a combination of high centrifugation speeds, low temperatures and variations in centrifugation cycles.^88^, ^89^ These conditions could induce premature activation of platelets during centrifugation, thereby affecting the regenerative capacities of the final PRP‐derived product.^86^ Therefore, research regarding PRP preparations should focus on the concentrations of PRP components, as well as on the optimal concentrations of platelets, leukocytes and growth factors for specific fields of application.^87^

Zheng *et al*. found that only optimal concentrations of PRP (<20%) produced the most beneficial effects with respect to stimulating cell proliferation, inducing the synthesis of neurotrophic factors and significantly increasing the migration of Schwann cells; moreover, such effects were dose‐dependent, and further increases in PRP concentration resulted in adverse effects.^90^ Giusti *et al*. determined that 1.5 × 10^6^ platelets/μL was the optimal concentration for the induction of angiogenesis in endothelial cells, whereas higher concentrations reduced the angiogenic potential of platelets with respect to follicular and perifollicular angiogenesis.^91^ Similarly, Graziani *et al*.^92^ assessed the biological rationale for the use of PRP by evaluating the effects of various concentrations of PRP on osteoblast and fibroblast function in vitro. They found that the maximum effect of cell proliferation in osteoblasts and fibroblasts was achieved at the final concentration of 16.5% (a platelet concentration of 2.5‐fold, relative to whole blood); greater concentrations reduced proliferation and had a suboptimal effect on cell function.

Many protocols have been established to identify the optimal characteristics of platelets, leukocytes and growth factors in a variety of clinical applications. These studies demonstrated that the preparation technique greatly influences platelet concentration and viability. Such variables affect the eventual concentration of bioactive molecules released from the platelet granules; moreover, they influence the clinical efficacies of individual PRP preparations.^93^

### Classification of PRP

Various classification systems have been established to standardize PRP, with the goal of better facilitating the interpretation of clinical studies.^94^ Ehrenfest *et al*. suggested that various platelet concentrates can be placed into four main families based on the separation of products using two key parameters: cellular content (primarily leukocytes) and fibrin architecture: (i) P‐PRP, (ii) L‐PRP, (iii) pure platelet‐rich fibrin (P‐PRF) and (iv) leukocyte‐and‐platelet‐rich fibrin (L‐PRF).^95^, ^96^, ^97^ This classification clearly defines the preparations based on leukocyte inclusion and fibrin clot presence while considering the facility and cost‐effectiveness involved with each system.^93^ In 2012, DeLong *et al*.^98^ published the ‘PAW’ classification system that recommended reporting PRP based on three components: the absolute number of platelets (P), the manner in which platelet activation occurs (A) and the presence or absence of white cells (W). Platelets were categorized as P1 (≤ baseline [i.e. concentration in whole blood]) to P4 (>1.2 × 10^6^ platelets/mL), activation as either exogenous (X) or not, and white blood cells and neutrophils as either above or below baseline. Overall, classification systems enable useful categorization of the important components of PRP, which can help direct clinicians’ therapeutic approaches. Therefore, such classifications must be carefully considered to avoid making incorrect conclusions when comparing results among clinical studies.^99^

## DISCUSSION

Regenerative therapies have been emerging as viable treatments for many diseases. The major therapeutic benefits of regenerative medicine treatments come from the paracrine action of trophic factors at significant concentrations which, among many actions, stimulate endogenous progenitor cells to promote proliferation and healing.^100^ PRP, serving as a vehicle and source of growth factors, is an autologous plasma preparation with concentrated platelets which has been extensively investigated for its application as a bioactive scaffold in cell‐based therapy and tissue engineering.^1^ In recent years, PRP has gradually become a focus in regenerative dentistry for its application in dentistry and oral surgery. Many studies have reported the efficacy of PRP as a treatment modality in different oral disorders.^11^, ^12^, ^101^, ^102^, ^103^ However, some concerns should be noted, particularly with regard to the timing of PRP treatment and the actual clinical effect on oral disorders, and methods of PRP preparation.^88^, ^104^

PRP has been suggested as a potential scaffold for regenerative endodontic therapy.^15^ However, in multiple preclinical animal studies and isolated clinical case reports, PRP induced the ingrowth of vascularized connective tissue in endodontically disinfected root canals, but showed minimal evidence of dentin formation.^16^ Thus, no current PRP products could act as scaffolds to regenerate the dentin–pulp complex.^17^ The ultimate goal of regenerative endodontics is the active pursuit of pulp and dentin regeneration through the application of tissue engineering technology. However, the development of this technology remains in early stages. Additional translational studies are needed to investigate the outcome of the application of PRP in regenerative endodontic treatment, as well as to establish a standardized PRP preparation protocol with respect to platelet concentration, type of clotting activator and leukocyte content with optimal ability to stimulate biological effects.^18^, ^109^

Published clinical reports have revealed a variety of controversial outcomes with respect to the therapeutic efficacy of PRP in periodontal regenerative procedures. Indeed, many factors can influence the results of periodontal regenerative therapy in these reports, including study design, graft materials, clinical parameters or observation period. The added effect of PRP when used in combination with different grafting materials has been controversial in a subset of controlled clinical studies.^110^ For example, some studies have shown greater improvements in PD reduction and CAL when PRP was combined with a grafting material, while others have failed to demonstrate significant differences.^27^, ^111^, ^112^, ^113^, ^114^, ^115^ Because of the slow metabolism of bone, radiographic bone filling may require a longer observation period to detect a positive result.^116^ Therefore, appropriate study design, careful determination of the surgical procedure and increased observation time should be considered when using PRP as an option for clinical periodontal regenerative treatment.

PRP was first introduced to the oral and maxillofacial surgery community by Whitman *et al*. in 1997.^117^ PRP contains many growth factors that can influence wound healing, implant placement and reconstructive surgery of mandibular defects.^118^ Significant improvements in local conditions are observed after PRP application. Thus far, studies have revealed that the high concentration of growth factors released in the alveolar socket after tooth extraction increases tissue regeneration and prevents the occurrence of local complications.^119^ Soft tissue healing is improved through the application of PRP, which increases collagen content, promotes angiogenesis and enhances early wound strength.^120^ PRP is also a valid technique for promoting bone regeneration at the distal surface of the mandibular second molar following extraction of impacted third molars.^121^ PRP reportedly improves bone regeneration in its early phases (i.e. between 3 and 6 weeks after oral surgery). Recently, *in vitro* investigations of the cellular mechanism underlying enhancement of bone repair have concluded that PRP stimulates chemotactic migration and proliferation of human mesenchymal cells in a dose‐dependent manner without loss of their potential for osteogenic development.^122^ However, some other studies on autologous growth factors demonstrated unfavourable results with promoting bone formation and healing. Ranly *et al*. reported that PRP treatment reduced osteoinductivity of demineralized bone matrix in immunocompromised mice.^123^ These results suggest that the majority of the reviewed clinical trials have reported encouraging outcomes, further properly controlled and well‐designed clinical trials are needed to provide solid evidence of the capacity of PRP for regenerative treatment in oral surgery.

Platelet‐rich concentrates such as PRP and PRF are recent innovations being utilized in injured dental tissue engineering. PRP is a concentrate of PRP protein obtained from whole blood and centrifuged to remove the red blood cells. The centrifuge speed and duration have been reported to influence platelet quantity, enrichment percentages, growth factor release and PRP efficacy.^124^ The double‐spin method of 160 *g* for 10 min followed by 250 *g* for 15 min resulted in increased platelet, cytokine and growth factor yields, and accelerated cellular migration and proliferation.^70^ In canines, centrifugation at 1000 *g* for 5 min and 1500 *g* for 15 min increased the platelet concentration by six times.^125^, ^126^ PRF is a second‐generation platelet‐rich concentrate where autologous platelets and leukocytes are present in a complex fibrin matrix. It was prepared from whole blood without addition of anticoagulants. Standard PRF is centrifuged at 3000 rpm for 10 min.^127^ The advantages of using PRF include ease of preparation and no addition of biochemical reagents or anticoagulants.^125^ Compared with PRP which becomes liquid end products that has short‐term effects, the PRF fibrin network forms a uniform three‐dimensional structure with long‐term effect on tissue regeneration by delivering cytokines slowly.^128^ PRP should be freshly prepared and used within 4 h, as almost 95% of the growth factors are secreted within the first hour after preparation. In contrast, PRF stimulates the microenvironment of tissue healing for a considerable period of time and continues to release growth factors up to 4 weeks.^129^, ^130^, ^131^ However, PRF is not capable of replacing PRP in all therapeutic areas, and its compact three‐dimensional structure hinders its use as an injectable agent.^132^ PRF is mainly used for replacing injured tissues in orthopaedics, and wound healing.^133^, ^134^ Moreover, a higher release of TGF‐β1, PDGF‐BB and VEGF was observed in PRP than in PRF in the first 8 h following PRP preparations. Specific platelet aggregation inhibition performed in PRP, but not in PRF, might be an important factor in growth factor yield.^79^, ^135^

Nowadays, abundance of commercially available PRP preparation systems claimed to yield consistent liquid end products and higher platelet counts than manual laboratory preparation.^136^ A literature reviewed 33 commercial PRP preparation systems, only 11 met the definition of 1 × 10^6^ platelets/μL as a minimum platelet concentration defined by Marx *et al*.^137^ Additionally, only 10 of the 33 systems analysed met the definition that platelets in PRP should be concentrated to at least five times that of baseline.^138^ The PRP yield differs in its qualities between different PRP preparation systems. Moreover, these commercial kits are costly.^139^ A commercial PRP preparation system costs from $US 175 to $US 1150 per kit, whereas the cost of a reliable manual PRP preparation could be less than 4 USD.

## CONCLUSIONS

In summary, as a biological surgical additive, PRP has been successfully used for various applications in dental regenerative medicine. However, some applications of PRP remain controversial. To further explore the clinical advantages of PRP, the generalized indications for its application and systematic preparation protocols should be established. Additional studies are needed to establish the therapeutic efficacy of PRP in regenerative dentistry; these studies should include randomized, controlled clinical trials designed to assess the long‐term benefits and ultimate outcomes of the application of PRP. Overall, PRP treatment appears to have a bright future in clinical regenerative dentistry.

## AUTHORS’ CONTRIBUTIONS

Jian Xu and Lingshan Gou wrote the manuscript. Peng Zhang, Hongwen Li and Shuqi Qiu provided critical comments on the manuscript.

## FUNDING STATEMENT

This work was supported by Shenzhen Health and Family Planning Committee of China (SZBC2017008) and The Innovation of Science and Technology Committee of Shenzhen Municipality (JCYJ20180305163353862 and JCYJ20180305163259711).

## CONFLICT OF INTEREST

The authors declare that there is no conflict of interest regarding the publication of this paper.
